# Identification of the effect and mechanism of Yiyi Fuzi Baijiang powder against colorectal cancer using network pharmacology and experimental validation

**DOI:** 10.3389/fphar.2022.929836

**Published:** 2022-10-24

**Authors:** Bin Xiang, Ruiman Geng, Zhengkun Zhang, Xuxu Ji, Jiaqiong Zou, Lihong Chen, Ji Liu

**Affiliations:** ^1^ Department of Biochemistry and Molecular Biology, West China School of Basic Medical Sciences and Forensic Medicine, Sichuan University, Chengdu, China; ^2^ Department of Laboratory Medicine, Clinical Medical College and the First Affiliated Hospital of Chengdu Medical College, Chengdu, China

**Keywords:** Yiyi Fuzi Baijiang powder, network pharmacology, colorectal cancer, inflammatory, SMOX

## Abstract

**Background:** Yiyi Fuzi Baijiang powder (YFBP) is a traditional Chinese medicine used to treat colorectal cancer, although its bioactivity and mechanisms of action have not been studied in depth yet. The study intended to identify the potential targets and signaling pathways affected by YFBP during the treatment of colorectal cancer through pharmacological network analysis and to further analyze its chemical compositions and molecular mechanisms of action.

**Methods:** The Traditional Chinese Medicine Systems Pharmacology (TCMSP), Traditional Chinese Medicine Integrated Database (TCMID), HitPredict (HIT), and Search Tool for Interactions of Chemicals (STITCH) databases were used to screen the bioactive components and promising targets of YFBP. Targets related to colorectal cancer were retrieved from the GeneCards and Gene Ontology databases. Cytoscape software was used to construct the “herb–active ingredient–target” network. The STRING database was used to construct and analyze protein–protein interactions (PPIs). Afterward, the R packages clusterProfiler and Cytoscape Hub plug-in were used to perform Gene Ontology (GO) functional and Kyoto Encyclopedia of Genes and Genomes (KEGG) pathway enrichment analyses of target genes. The results of the network pharmacological analysis were also experimentally validated.

**Results:** In total, 33 active components and 128 target genes were screened. Among them, 46 target genes were considered potential therapeutic targets that crossed the CRC target genes. The network pharmacology analysis showed that the active components of YFBP were correlated positively with CRC inflammatory target genes such as TLR4, TNF, and IL-6. The inflammation-related signaling pathways affected by the active components included the TNF-α, interleukin-17, and toll-like receptor signaling pathways. The active ingredients of YFBP, such as luteolin, β-sitosterol, myristic acid, and vanillin, may exert anti-tumor effects by downregulating SMOX expression *via* anti-inflammatory signaling and regulation of the TLR4/NF-κB signaling pathway.

**Conclusion:** In the present study, the potential active components, potential targets, and key biological pathways involved in the YFBP treatment of CRC were determined, providing a theoretical foundation for further anti-tumor research.

## Background

Cancer is a major public health issue and is the main cause of death worldwide (Sung et al., 2021). It is a multifactorial disease affected by both environmental and genetic factors that also impacts life expectancy (Bray et al., 2021). Colorectal cancer (CRC) is ranked third in the incidence rate and second in mortality among the top 10 most common cancer types worldwide in 2020 (Sung et al., 2021). CRC is a malignancy that develops in the colon and rectum and is a common gastrointestinal malignancy with a poor prognosis in the advanced stages (Chen et al., 2020). CRC, like other cancers, is affected by genetic, environmental, and lifestyle factors, including deregulation of the microbiota and diseases such as obesity and type 2 diabetes (Zagato et al., 2020). The etiology and pathogenesis of CRC are unclear but are related to risk factors such as high-fat and low-fiber diets. Poor diet and lifestyle are directly related to 80% of CRC incidence (Vargas and Thompson, 2012).

Polyamines such as putrescine, spermidine, and spermine are small alkaline molecules containing two or more primary amino groups; they participate in a variety of cellular functions, including protein synthesis, DNA and RNA structure, protein–RNA interactions, and gene expression (Luo et al., 2009; Miller-Fleming et al., 2015; Pegg, 2016; Olsen et al., 2018). Studies have shown that polyamine metabolism is dysregulated in many types of cancer (Casero and Marton, 2007). Ultimately, an imbalance in polyamine metabolism causes cellular injury and tissue damage. Polyamine levels are significantly increased during carcinogenesis and are involved in tumor transformation and regulation of the tumor microenvironment, as such, polyamine regulation is closely associated with the occurrence and development of tumors and is therefore an attractive potential therapeutic target for anti-tumor therapy (Daniel et al., 2017; Casero et al., 2018).

The metabolism of natural polyamines is regulated by spermine oxidase (SMOX), spermine/spermidine N1-acetyltransferase (SSAT), and polyamine oxidase (APAO) (Xie et al., 2017). SMOX, an inducer of polyamine catabolism, is found both in the cytoplasm and nucleus and is responsible for the direct reverse conversion of spermine to spermidine (Murray-Stewart et al., 2016). It catalyzes the oxidative degradation of spermine to produce aldehyde 3-aminopropanal, hydrogen peroxide, and spermidine (Wang et al., 2001). SMOX expression can rapidly be induced at dramatically different levels in response to various stimuli, including natural polyamines, pro-inflammatory cytokines, bacterial infections, and certain polyamine analogs (Babbar and Casero, 2006; Goodwin et al., 2011). The upregulated expression of SMOX can increase local reactive oxygen species (ROS) and trigger a DNA-damage response, leading to stress-induced or injury-induced CRC, respectively (Xu et al., 2004). Because of this, elevated SMOX levels are a molecular link between inflammatory stimuli, infection, and chronic inflammation-related tumorigenesis. Numerous studies have shown that SMOX activity can be inhibited without deleterious effects (Seiler et al., 2002; Babbar and Casero, 2006). It is important to understand the potential application of small-molecule inhibitors specifically targeting SMOX for furthering the prevention and treatment options for diseases such as CRC.

For thousands of years, natural products and their derivatives have played an important role in disease research and treatment (Gerry and Schreiber, 2018). Yiyi Fuzi Baijiang powder (YFBP), a classic traditional Chinese medicine, is widely used for the prevention and treatment of many disorders. It is derived from a famous formula recorded in the “Synopsis of Golden Chamber” by Zhongjing Zhang during the Han Dynasty, consisting of 30 g Coicis Semen, 6 g Aconiti Lateralis Radix Praeparata, and 15 g Herba Patriniae. According to books on ancient Chinese medicine, YFBP was used to clear heat, remove dampness and purulent discharge, and for detumescence. YFBP is a representative prescription for the treatment of intestinal carbuncles.

Network pharmacology, which is based on a disease–gene–drug network, is a promising new approach in current drug discovery and development, especially in traditional Chinese medicine research (Shen et al., 2020).

**FIGURE 1:**
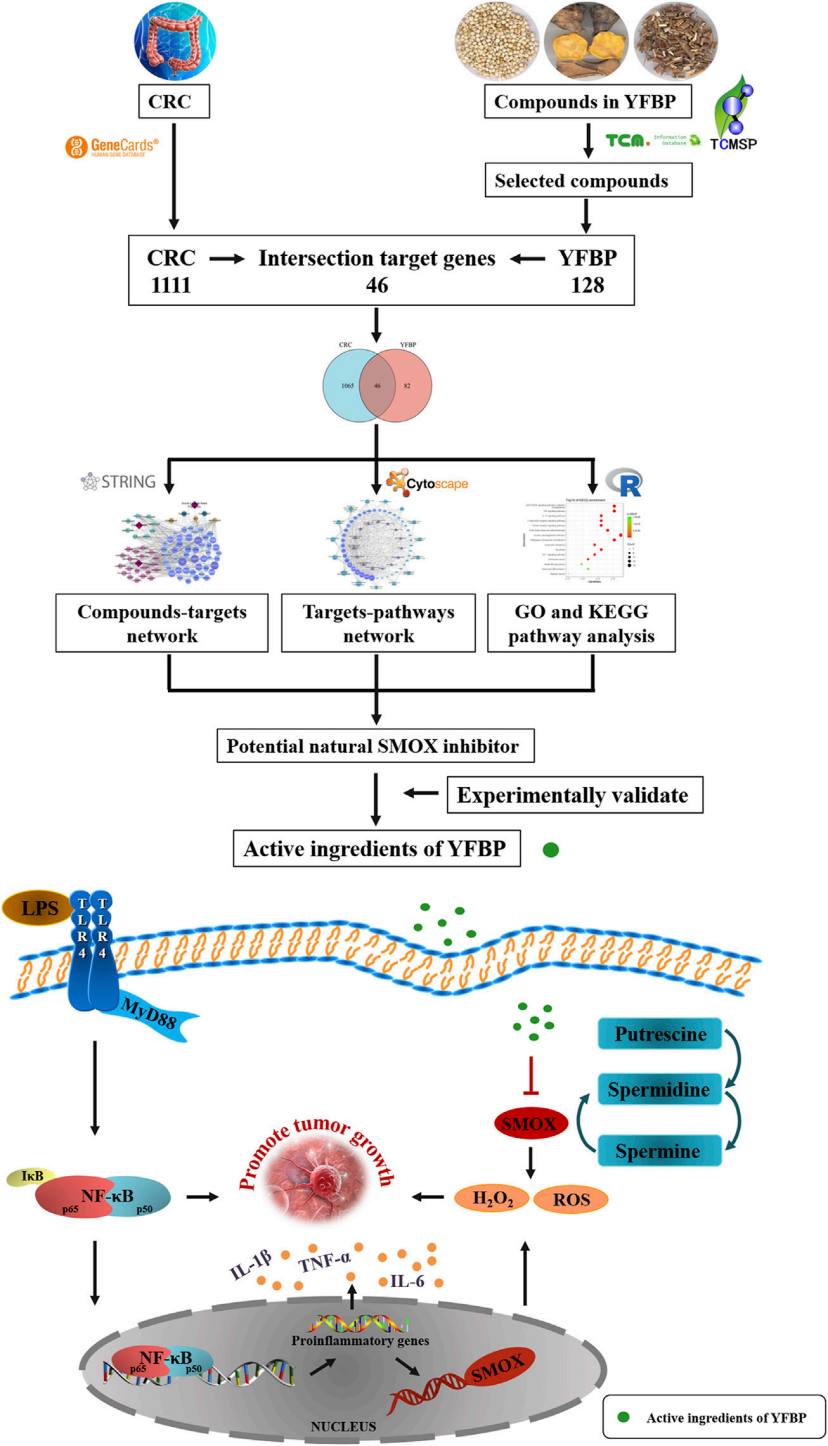
Working flow chart of SMOX inhibitor prediction in CRC.

In the present study, a network pharmacology approach was used to predict the molecular targets and bioactive ingredients of YFBP. The active components of YFBP were screened for the first time, and the CRC-related targets of the active components were analyzed and summarized. Gene Ontology (GO) and biological pathway (KEGG) enrichments were used to study the targets and mechanistic pathways impacted by the active ingredients. The results of network pharmacological analysis were also experimentally validated. Overall, this study aimed to provide a theoretical basis for the molecular mechanism of action of YFBP during CRC (Figure 1).

## Materials and methods

### Analysis of YFBP by network pharmacology

#### Candidate compound screening

All chemical constituents of YFBP were collected from the Traditional Chinese Medicine Systems Pharmacology (TCMSP) database (Ru et al., 2014), Traditional Chinese Medicine Integrated Database (TCMID) (Huang et al., 2018), and HitPredict (HIT) database (Ye et al., 2011), and target-free compounds were eliminated.

#### Potential disease targets of YFBP

The GeneCards database was used to identify potential targets implicated in CRC. All target compounds were collected from the TCMSP, TCMID, HIT, and Search Tool for Interactions of Chemicals (STITCH) databases. A Venn diagram was used to determine the main targets of YFBP in CRC treatment. The protein–protein interaction (PPI) network analysis was performed using the STRING database.

#### Pathway and functional enrichment analysis

GO and KEGG were used to analyze signaling pathways and biological processes related to the target CRC-related genes. GO functional and KEGG pathway enrichment analyses of target genes were performed using the R package clusterProfiler and Cytoscape Hub plug-in.

#### Network construction analysis

All network plots were constructed using Cytoscape software. Cytoscape with an in-house plugin was used to evaluate and screen core targets to form a core network that was formed to assess the relationship of KEGG-associated items of YFBP and CRC.

### Experimental verification

#### Cell culture and viability assay

**FIGURE 2:**
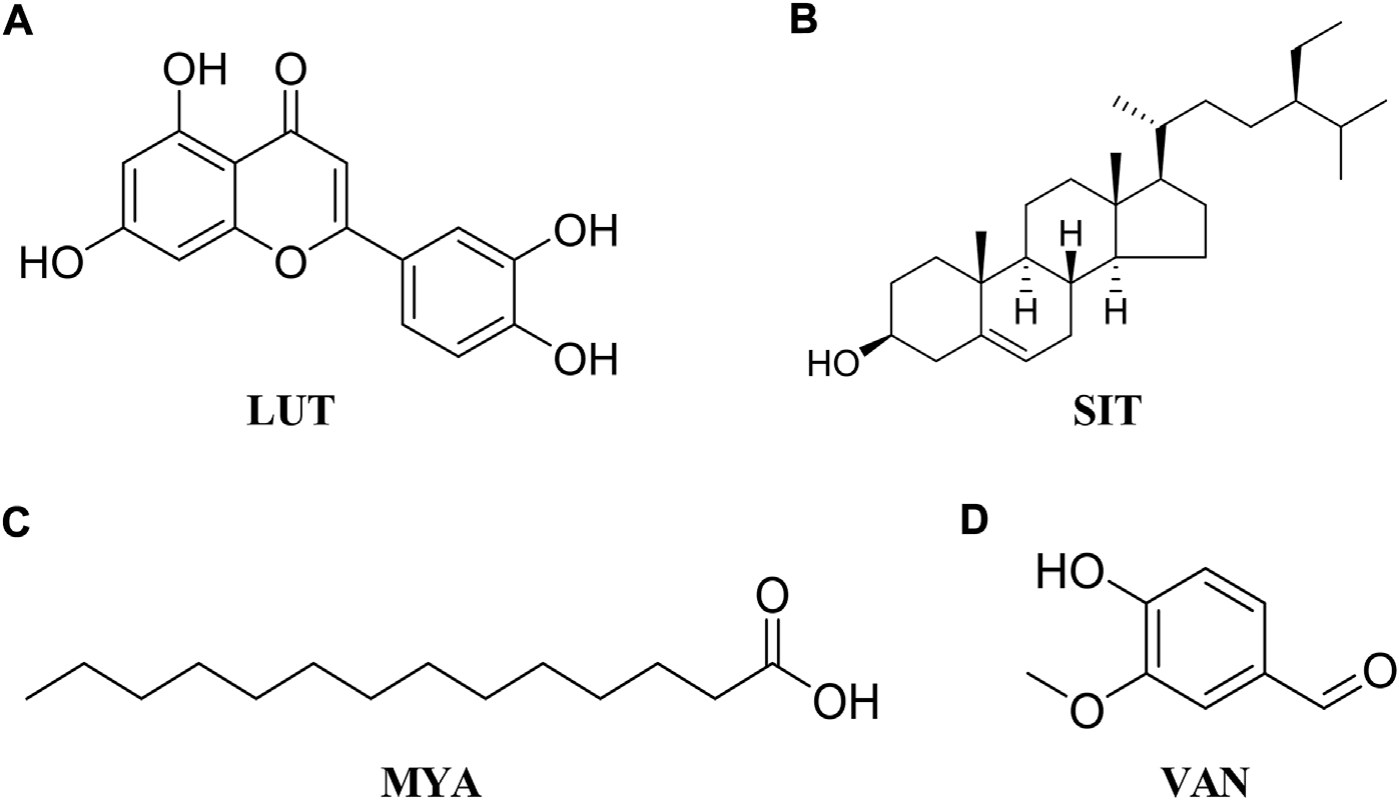
Chemical structure of LUT **(A)**, SIT **(B)**, MYA **(C)**, and VAN **(D)**.

HT-29 cells were purchased from Jiangsu KeyGEN BioTECH Corp. (Jiangsu, China). The cells were cultured at 37°C and 5% CO_2_ in DMEM 1640 (Gibco) that contained 10% fetal bovine serum (FBS, Gibco) and 1% penicillin–streptomycin (Gibco). When the cells reached 80% confluence, they were divided and sub-cultured for further passages with 0.25% trypsin and 0.02% ethylene-diamine-tetra-acetic acid (EDTA, Gibco). Cell viability was determined using the cell counting kit-8 (CCK-8) assay (APExBIO Technology LLC, United States). Briefly, cells were seeded and treated with different concentrations of luteolin (LUT, Aladdin, Figure 2A), β-sitosterol (SIT, Aladdin, Figure 2B), myristic acid (MYA, J&K Scientific, Figure 2C), and vanillin (VAN, J&K Scientific, Figure 2D) for 24 h, and 10% CCK-8 solution was added to each well. After incubation for 2 h at 37°C, the absorbance at 490 nm was read on a microplate reader (iMark, Bio-Rad, United States).

#### HPLC analysis

High-performance liquid chromatography (HPLC, LC-2030C Plus, Shimadzu Corporation, Japan) was used to measure the content of LUT, SIT, MYA, and VAN in YFBP. A volume of 51.00 g of YFBP was accurately weighed and 100 ml of methanol was added, and the mixture was ultrasonicated for 12 h at 60°C. The extracts were filtered using a filter paper and concentrated to 1.00 g crude drug per ml. The concentrated solution was taken and passed through a 0.22-µm microporous membrane, and the content of LUT, SIT, MYA, or VAN was determined by HPLC. LUT, SIT, MYA, or VAN standards were accurately weighed and dissolved in methanol as the stock solution. Chromatography was performed on a C18 column (5 μm C18, 4.6 × 250 mm) at 35°C. LUT was eluted with a mobile phase (methanol/0.1% phosphoric acid water at a ratio of 50:50) at a flow rate of 0.5 ml/min and monitored at 350 nm. SIT was eluted with a mobile phase (methanol/water at a ratio of 55:45) at a flow rate of 0.5 ml/min and monitored at 210 nm. MYA was eluted with a mobile phase (acetonitrile/water at a ratio of 33:67) at a flow rate of 0.5 ml/min and monitored at 270 nm. VAN was eluted with a mobile phase (acetonitrile/0.1% phosphoric acid water at a ratio of 13:87) at a flow rate of 0.5 ml/min and monitored at 281 nm. The chromatograms were recorded and integrated using LabSolutions System software.

#### Cell scratch, invasion, and apoptosis assays

Cell scratch, invasion, and apoptosis assays were performed as described previously (Choe et al., 2018; Mazzu et al., 2019). Briefly, for cell scratch assay, cells were seeded in six-well plates for 48 h. A scratch was made using a pipette tip in a confluent area. The assay was conducted in triplicate under each condition. Photographs of each scratch were taken at 24 and 48 h after scratching. For cell invasion assay, the number of traversing cells was measured using the Transwell Matrigel Invasion Chamber (Corning, United States) following the manufacturer’s instructions. For apoptosis assay, the numbers of apoptotic cells were quantified by flow cytometric assays using the Annexin V-FITC/PI Apoptosis Detection Kit (Jiangsu KeyGEN BioTECH Corp., Ltd) 24 h after the treatment of 20 μg/ml of LUT, SIT, MYA, or VAN and cell seeding.

#### Animals

SPF male BALB/c nu/nu mice, weighing 18–22 g (West China University Second Hospital, Sichuan University, Chengdu, China), were housed in an animal laboratory at 25°C and 60% humidity. HT-29 cells in the logarithmic growth phase were selected and digested to prepare a single-cell suspension with a density of 1×10^7^/ml. A total of 2×10^6^ HT-29 cells were subcutaneously injected into the back of the nude mice. Two weeks after tumor formation, qualified nude mice were randomly divided into five groups: control (CTR), LUT (50 mg/kg), SIT (50 mg/kg), MYA (50 mg/kg), and VAN (50 mg/kg). For each drug, the administration groups were given the drug for 21 days (q.d.) after tumor formation. After 21 days of continuous administration, the nude mice were sacrificed, and tumors were isolated for further experimental study.

All experimental protocols and animal handling procedures were performed in accordance with the Experimental Animal Ethics Committee of Sichuan University.

### Histological staining

Hematoxylin/eosin (HE) staining of tumor samples followed standard HE staining procedures. The tumor tissue was cut off and fixed in 4% polymethylaldehyde solution for 24 h. After alcohol gradient dehydration, about 6-μm-thick coronal sections were embedded in transparent paraffin, and dewaxing was performed with xylene. Each tissue section was incubated at 62°C for 24 h. After thorough dewaxing, the slide with the tissue sections was passed through the decreasing concentration of alcohol to remove xylene I and II and thoroughly rinsed in water. The slide was stained with a hematoxylin staining solution, and for the differentiation step, 1% hydrochloric acid alcohol was used to remove non-specific background staining and to improve contrast. After differentiation, blueing and thorough rinsing with water were performed. For counterstaining, 5% eosin was used. After counterstaining, the slide was passed through 80%, 95%, and 100% alcohol to remove all water traces and rinsed with xylene, which clears the tissue and renders it completely transparent. The slide was sealed with neutral gum. Optical microscopic random observations of the tumor tissue cortex at ×400 magnification do not overlap the three fields of vision and radiography.

### Immunohistochemistry

The tumor tissue was excised and fixed in 4% polymethylaldehyde solution for 24 h. All the samples were paraffin-embedded and sectioned. Immunostaining for SMOX was performed in tumor slices using rabbit anti-SMOX primary antibodies (1:500; Proteintech, United States).

### ELISA

The level of SMOX in the serum obtained from BALB/c nu/nu mice 21 days after tumor formation was measured using an ELISA kit (Cloud-Clone Corp, Wuhan, China), according to the manufacturer’s instructions.

### Quantitative real-time PCR analysis

The qRT-PCR analysis was performed as described in a previous study (Li et al., 2020). Briefly, gene expression of pro-inflammatory cytokines (tumor necrosis factor-α (TNF-α), interleukin-1β (IL-1β), and interleukin-6 (IL-6)) and proteins (SMOX, TLR4, NF-κB p65, and p-NF-κB p65) was evaluated in the tumor tissue obtained from BALB/c nu/nu mice 21 days after tumor formation. Total RNA was isolated using TRIzol reagent (Nanjing Vazyme Biotech Co., Ltd.). A volume of1 μg of total RNA was reverse-transcribed using SuperScript III transcriptase (Nanjing Vazyme Biotech Co., Ltd.). Quantitative real-time PCR (qRT-PCR) was conducted using a qTOWER (Chen et al., 2020) G system (Analytik Jena, Germany) with SYBR Green to determine the mRNA expression level of the gene of interest. Expression levels were normalized to GAPDH (see Table 1 for detailed sequences). Measurements were performed in triplicate, and the results were analyzed using the 2^−ΔΔCt^ method for relative quantification. A list of primers designed for qRT-PCR is shown in Table 1.

**TABLE 1:** List of primers designed for qRT-PCR.

Primer name	Primer sequence	
SMOX	Forward	5′- ACGGAGATGCTGCGTCAGTTCA -3′
	Reverse	5′- CCTGCGTGTATGAATAGGAGCC -3′
TNF-α	Forward	5′-CTCTTCTGCCTGCTGCACTTTG -3′
	Reverse	5′- ATGGGCTACAGGCTTGTCACTC -3′
IL-1β	Forward	5′- CCACAGACCTTCCAGGAGAATG -3′
	Reverse	5′- GTGCAGTTCAGTGATCGTACAGG -3′
IL-6	Forward	5′-AGACAGCCACTCACCTCTTCAG -3′
	Reverse	5′- TTCTGCCAGTGCCTCTTTGCTG -3′
TLR4	Forward	5′-CCCTGAGGCATTTAGGCAGCTA-3′
	Reverse	5′-AGGTAGAGAGGTGGCTTAGGCT-3′
NF-κB p65	Forward	5′-TGAACCGAAACTCTGGCAGCTG-3′
	Reverse	5′-CATCAGCTTGCGAAAAGGAGCC-3′
GAPDH	Forward	5′- GTCTCCTCTGACTTCAACAGCG -3′
	Reverse	5′-ACCACCCTGTTGCTGTAGCCAA -3′

### Western blot analysis

Western blotting was performed as described in a previous study (Xiang et al., 2018). Briefly, after various treatment protocols, the tumor tissues were lysed with RIPA buffer, and proteins were extracted. A BCA kit (Beyotime Biotechnology) was used to determine the protein concentration. Sodium dodecyl sulfate-polyacrylamide gel electrophoresis was used to separate the proteins, which were then transferred to a polyvinylidene difluoride membrane and incubated with primary antibodies. The following primary antibodies were used: SMOX (1:1000), TLR4 (1:1000), NF-κB p65 (1:1000), phosphor (p)-NF-κB p65 (1:500), and β-actin (1:1000) (all purchased from Proteintech, United States). After incubation, appropriate secondary antibodies (1:2000; Proteintech, United States) were selected and incubated with the membrane before the immune complexes were observed using a chemiluminescence kit. Quantity one was used to obtain the relative gray value of the signal.

### Chromatin immunoprecipitation assays

The chromatin immunoprecipitation (ChIP) experiment was performed as previously described (Ye et al., 2019). Briefly, cells were seeded and incubated as mentioned previously for 24 h. After adding LUT, SIT, MYA, or VAN, the cells were further cultured for 24 h. ChIP was performed using the ChIP assay kit (Beyotime Institute of Biotechnology, Beijing, China) to test whether the transcription factor NF-κB p65 binds to SMOX, according to the manufacturer’s instructions. Western blot analysis was performed to confirm the successful immune precipitation of the transcription factor NF-κB p65 which binds to SMOX in colorectal cancer cells.

### Statistical analysis

Data are presented as mean ± SEM (standard error of the mean). All data were analyzed by ANOVA using SPSS. The levels of significance were set at *p < 0.05 and **p < 0.01.

## Results

### YFBP active ingredient database establishment

A total of 81 YFBP components were identified from the TCMSP, TCMID, and HIT databases. Based on the quantitative estimate of the drug-likeness (QED) value of drug bank-listed compounds, 0.2 was selected as the threshold for screening (Bickerton et al., 2012). In total, 33 YFBP active components were screened for database establishment (Table 2). The active components included quercetin, scopoletin, kaempferol, FER, apigenin, esculetin, palmitic acid, luteolin, ursolic acid, acetic acid, stigmasterol, α-humulene, acacetin, isorhamnetin, oleanolic acid, beta-sitosterol, higenamine, CAM, limonene, perillyl alcohol, hirsutrin, isoorientin, inositol, vanillin, EIC, oleic acid, CLR, hexanal, myristic acid, stearic acid, linolenic acid, nonanoic acid, and caprylic acid.

**TABLE 2:** Characteristics of active components in YFBP.

No.	Molecule name	QED	No.	Molecule name	QED
1	quercetin	**0.51**	**18**	CAM	**0.52**
2	scopoletin	**0.54**	**19**	limonene	**0.48**
3	kaempferol	**0.64**	**20**	perillyl alcohol	**0.6**
4	FER	**0.72**	**21**	hirsutrin	**0.27**
5	apigenin	**0.74**	**22**	isoorientin	**0.29**
6	esculetin	**0.36**	**23**	inositol	**0.24**
7	palmitic acid	**0.37**	**24**	vanillin	**0.52**
8	luteolin	**0.6**	**25**	EIC	**0.29**
9	ursolic acid	**0.44**	**26**	oleic acid	**0.2**
10	acetic acid	**0.42**	**27**	CLR	**0.49**
11	stigmasterol	**0.46**	**28**	hexanal	**0.29**
12	alpha-humulene	**0.49**	**29**	myristic acid	**0.45**
13	acacetin	**0.89**	**30**	stearic acid	**0.3**
14	isorhamnetin	**0.67**	**31**	linolenic acid	**0.33**
15	oleanolic acid	**0.45**	**32**	nonanoic acid	**0.58**
16	beta-sitosterol	**0.44**	**33**	caprylic acid	**0.58**
17	higenamine	**0.63**			

### Screening of potential YFBP bioactive compounds and candidate targets

**FIGURE 3:**
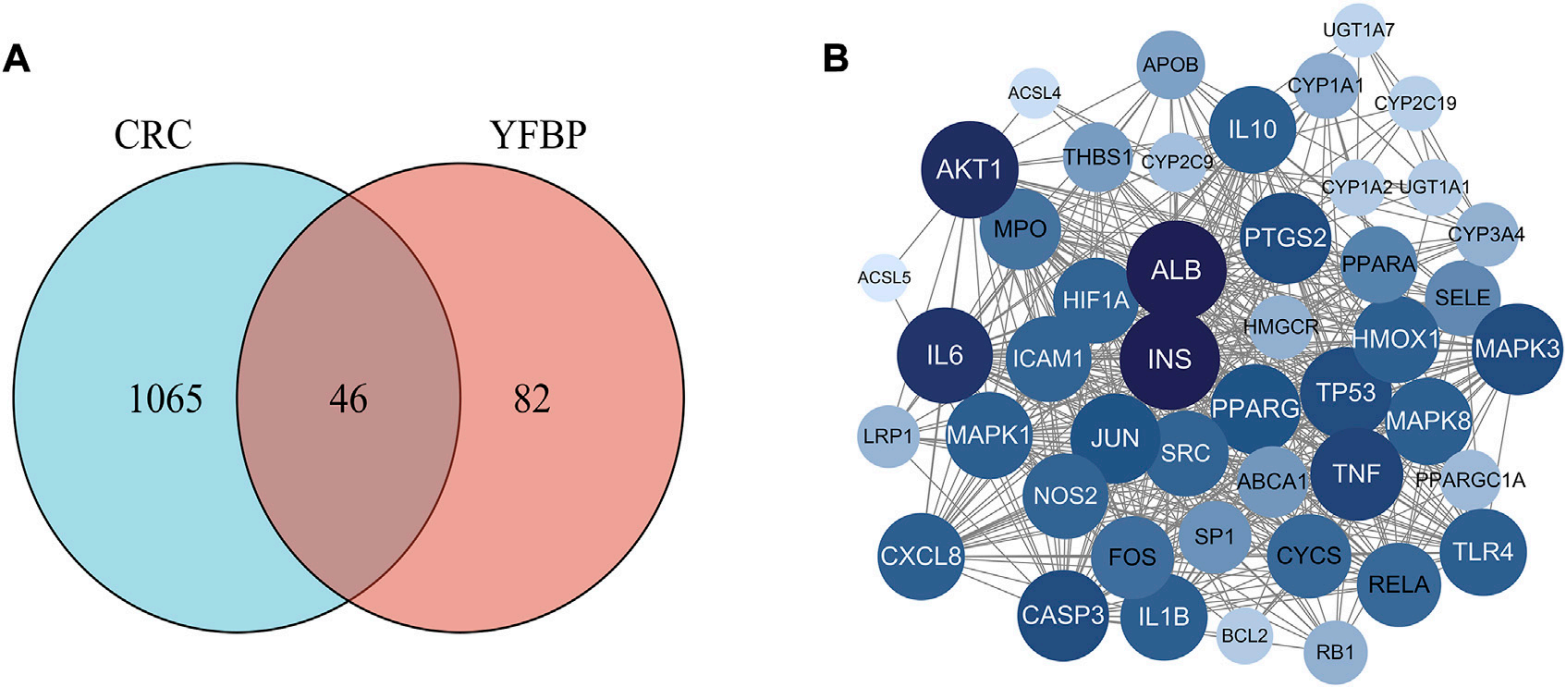
Potential target genes and the PPI network map of YFBP against CRC. **(A)** Venn diagram of potential target genes of YFBP against CRC. **(B)** PPI network map of 46 target genes.

Based on the GeneCards database (Rappaport et al., 2017), a total of 1,111 related targets were obtained with suspected or confirmed involvement in colorectal cancer. A total of 46 potential target genes were identified using Venny software to cross-target genes involved in CRC with those affected by YFBP (Figure 3A; Table 3). The PPI network map of the 46 target genes was then obtained using the STRING database (Figure 3B). A total of 46 nodes and 516 lines were obtained from the PPI network map, with an average degree value of 22.4.

**TABLE 3:** 46 potential target genes on YFBP against CRC.

No.	Entrez ID	Symbol	No.	Entrez ID	Symbol
1	3725	JUN	**24**	3569	IL6
2	19	ABCA1	**25**	3630	INS
3	2182	ACSL4	**26**	4035	LRP1
4	51703	ACSL5	**27**	5594	MAPK1
5	207	AKT1	**28**	5595	MAPK3
6	213	ALB	**29**	5599	MAPK8
7	338	APOB	**30**	4353	MPO
8	596	BCL2	**31**	4843	NOS2
9	836	CASP3	**32**	5465	PPARA
10	3576	CXCL8	**33**	5468	PPARG
11	54205	CYCS	**34**	10891	PPARGC1A
12	1543	CYP1A1	**35**	5743	PTGS2
13	1544	CYP1A2	**36**	5970	RELA
14	1557	CYP2C19	**37**	6401	SELE
15	1559	CYP2C9	**38**	6667	SP1
16	1576	CYP3A4	**39**	6714	SRC
17	2353	FOS	**40**	7057	THBS1
18	3091	HIF1A	**41**	7099	TLR4
19	3156	HMGCR	**42**	7124	TNF
20	3162	HMOX1	**43**	7157	TP53
21	3383	ICAM1	**44**	54658	UGT1A1
22	3586	IL10	**45**	54577	UGT1A7
23	3553	IL1B	**46**	5925	RB1

### Construction and analysis of the YFBP-CRC potential target gene network

**FIGURE 4:**
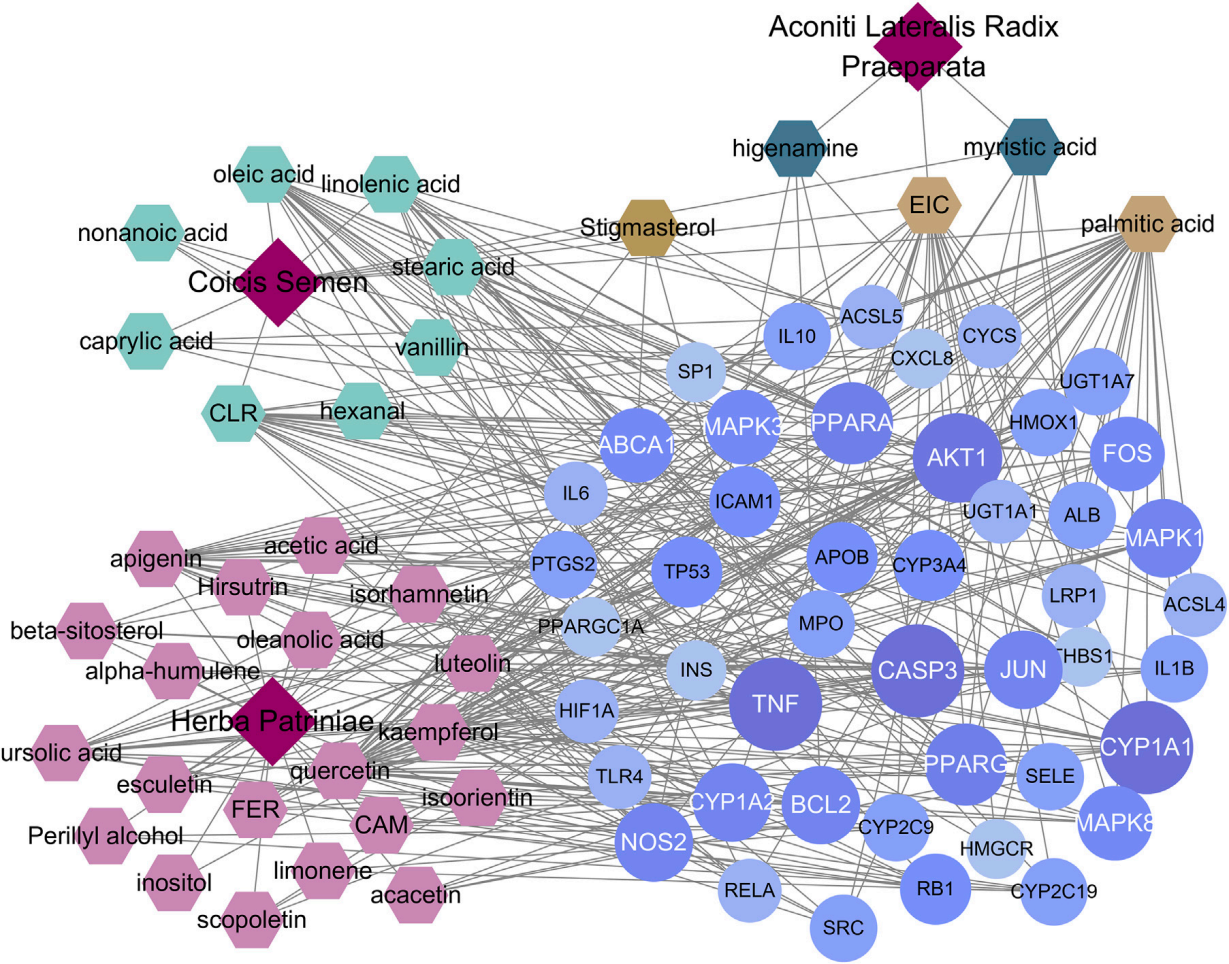
YFBP-CRC potential target gene network. The size of each node and the color depth in the network represent the size of its degree.

The results of 33 active ingredients and 46 potential target genes were entered into Cytoscape software to obtain a YFBP-CRC potential target gene network (Figure 4). The violet circle in the figure represents potential targets of YFBP therapy for CRC. The size of each node and the color depth in the network represent the size of its degree. The gray connecting lines indicate that each node is connected to each other. Hexagons represent compounds, different colors represent compounds in different traditional Chinese medicines, earthy yellow represents the compounds contained in more than two traditional Chinese medicines, and red quadrilateral represents different traditional Chinese medicines.

### GO and KEGG pathway enrichment analyses

**FIGURE 5:**
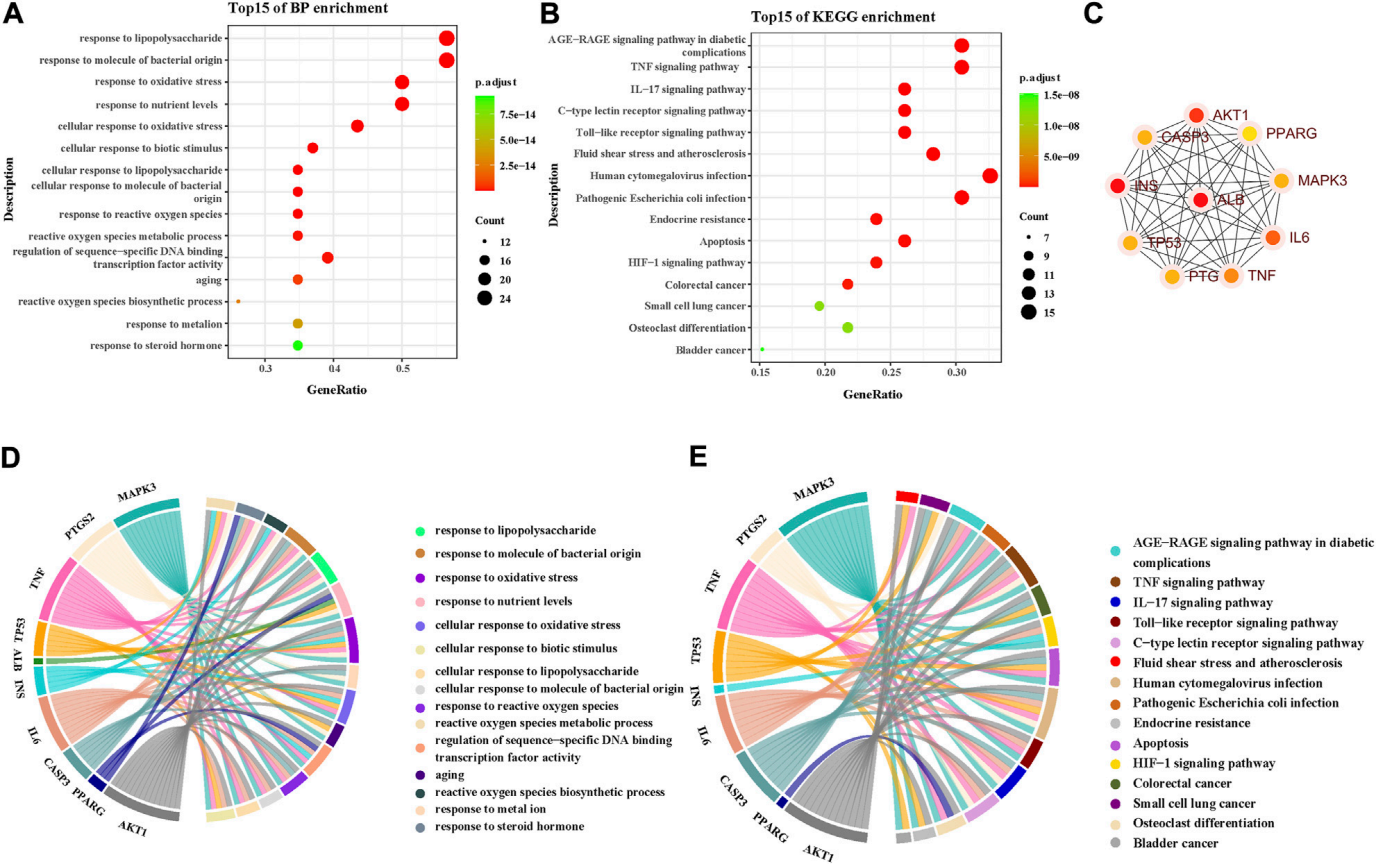
GO and KEGG pathway enrichment analyses. **(A)** Top 15 significantly rich GO analyses for the biological function of potential target genes of YFBP in CRC. **(B)** KEGG analysis of the first 15 significantly abundant YFBP potential target gene signaling pathways in CRC. **(C)** Top 15 vital signaling pathways’ core network. **(D)** Correlation between the core network and the top 15 significantly abundant gene biological function catalogs. **(E)** Correlation between the core network and the top 15 vital signaling pathways.

A total of 615 related biological functions were obtained by the GO enrichment analysis (*p* < 0.05, single channel count ≥5). A scatter plot was generated by selecting 15 distinct enriched gene biological function catalogs (Figure 5A). The biological functions of these genes mainly included response to lipopolysaccharides, response to molecules of bacterial origin, response to oxidative stress, response to nutrient levels, cellular response to oxidative stress, cellular response to biotic stimulus, cellular response to lipopolysaccharides, cellular response to molecules of bacterial origin, response to ROS, ROS metabolic process, regulation of sequence-specific DNA-binding transcription factor activity, aging, ROS biosynthetic process, response to metal ions, and response to steroid hormones. The core network was composed of 10 YFBP target genes with a high degree of potential for involvement in CRC (Figure 5C). The correlation between the core network and the top 15 significantly rich catalogs of gene biological functions is shown in Figure 5D.

To further understand the mechanism of YFBP treating CRC, 100 signaling pathways were identified by the KEGG pathway enrichment analysis (*p* < 0.05, single channel count ≥5). A scatter diagram (Figure 5B) was made using the first 15 important signaling pathways. Figure 5B shows that many signaling pathways are closely related to CRC, such as the AGE-RAGE in diabetic complications, TNF, IL-17, toll-like receptors, C-type lectin receptors, fluid shear stress, atherosclerosis, human cytomegalovirus infection, pathogenic *Escherichia coli* infection, endocrine resistance, apoptosis, HIF-1 signaling pathway, colorectal cancer signaling pathway, small-cell lung cancer signaling pathway, osteoclast differentiation, and bladder cancer signaling pathway. The correlation between the core network and the top 15 vital signaling pathways is shown in Figure 4E.

**FIGURE 6:**
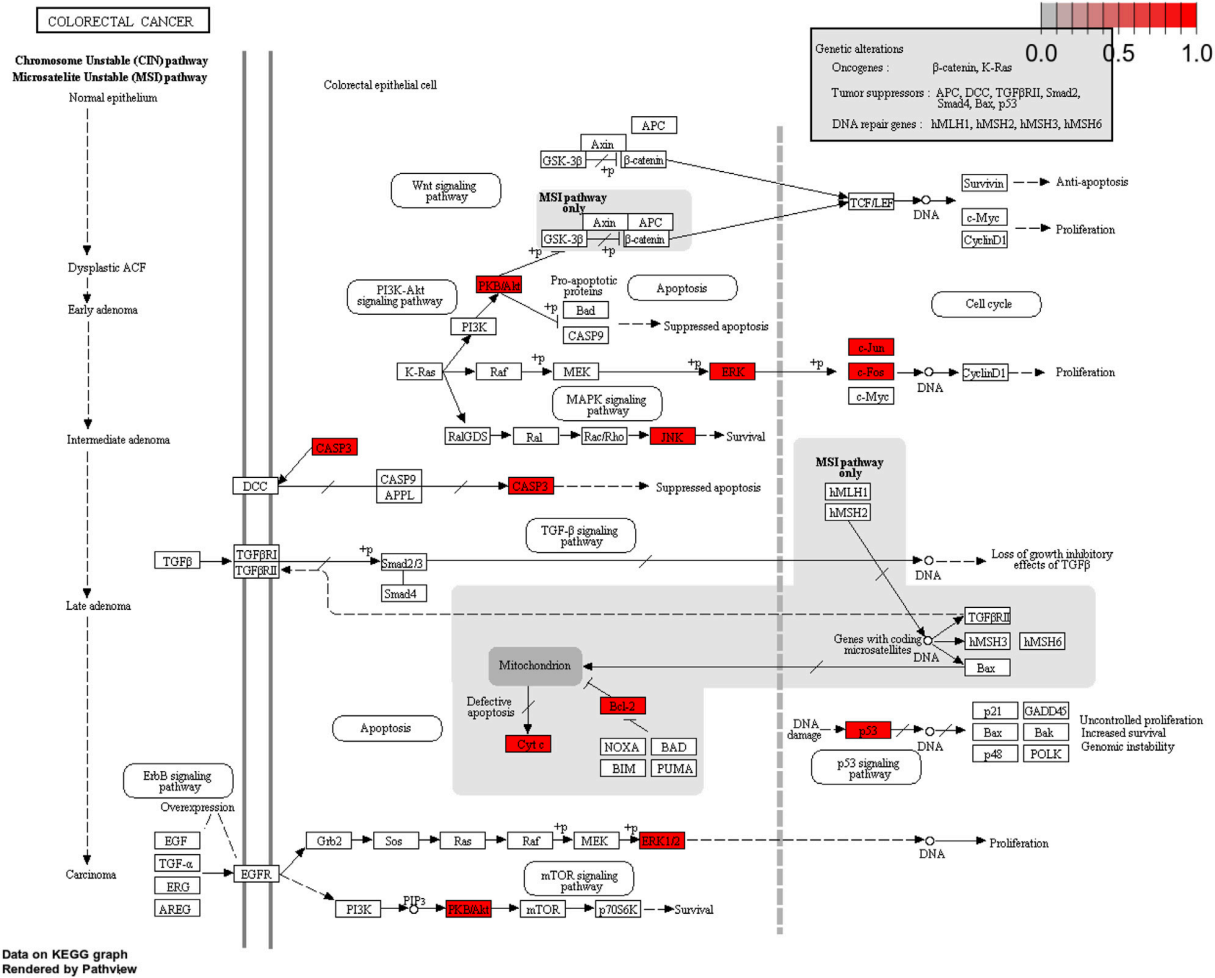
Colorectal cancer signaling pathway of YFBP potential target genes on CRC. The red represents YFBP target genes in the network.

**FIGURE 7:**
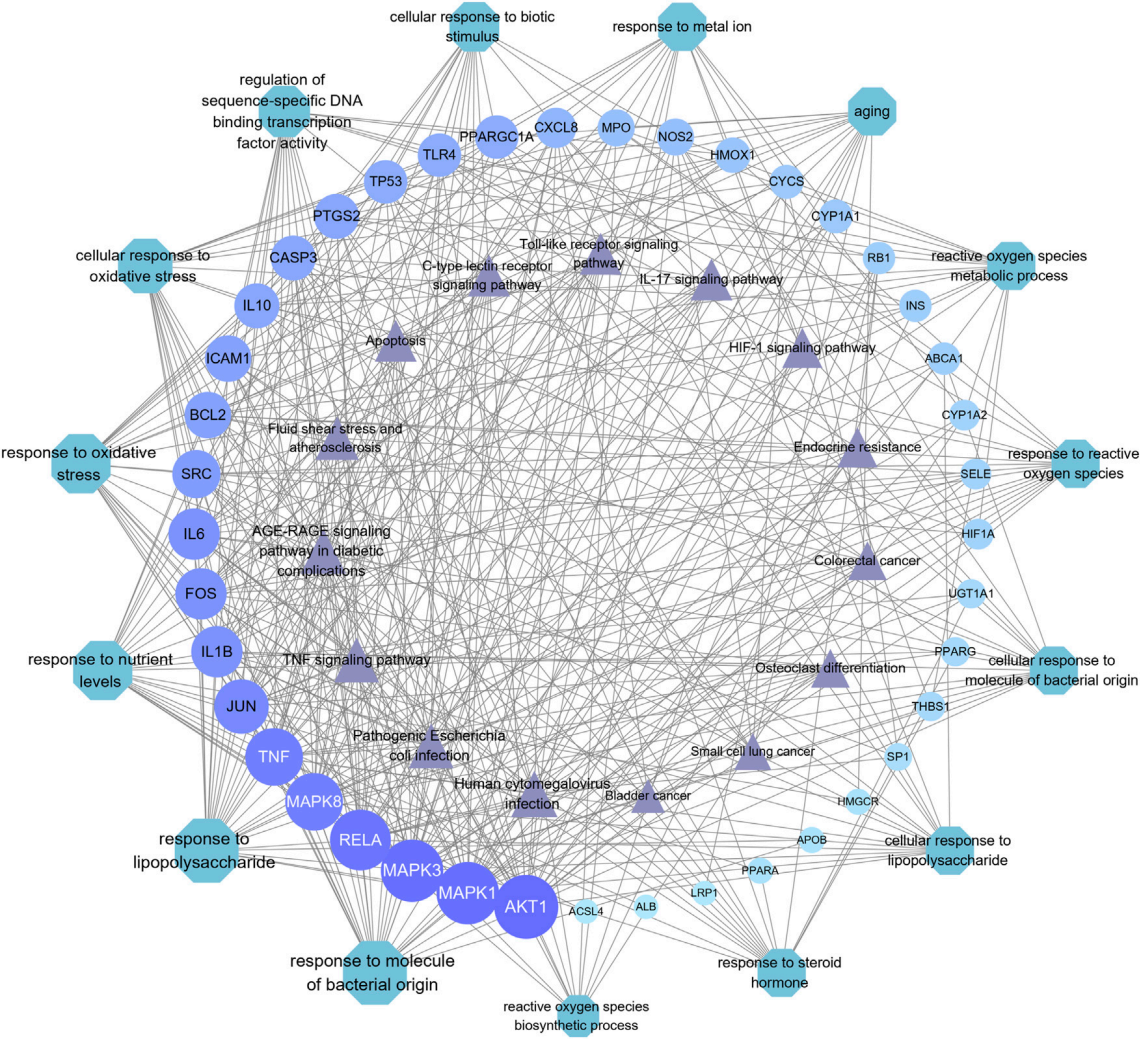
Correlation between core target genes, the top 15 biological function catalogs, and the top 15 signaling pathways. The size of each node and the color depth in the network represent the size of its degree. Each node is interconnected by the gray connection line.

The important CRC signaling pathways are shown in Figure 6. The correlations between potential target genes of YFBP in CRC therapy, the top 15 significantly rich catalogs of gene biological functions, and the top 15 vital signaling pathways are shown in Figure 7. The results showed that YFBP interacts with CRC through a variety of biological functions and signaling pathways, which is helpful for understanding the anti-inflammatory mechanisms affected by the YFBP treatment of CRC.

### HPLC measurements of LUT, SIT, MYA, and VAN concentrations in YFBP

**FIGURE 8:**
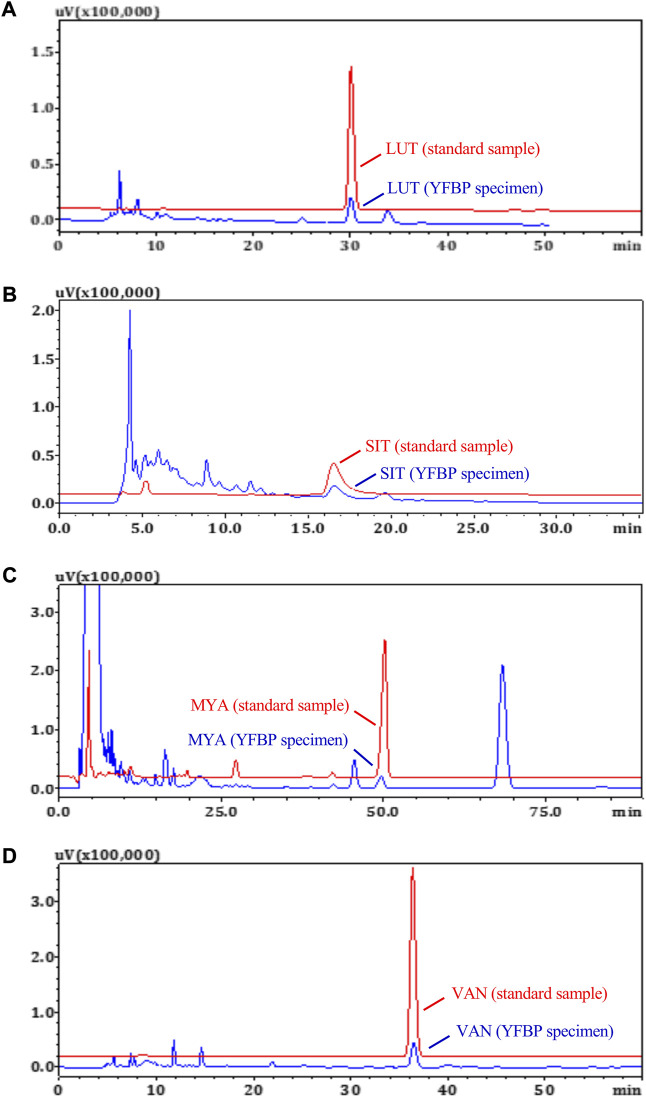
HPLC measurements of LUT **(A)**, SIT **(B)**, MYA **(C)**, and VAN **(D)** concentrations in YFBP. Red represents the standard, and blue represents the sample.

The content of LUT, SIT, MYA, and VAN in YFBP was measured by HPLC. LUT, SIT, MYA, or VAN was monitored at 350 nm, 210 nm, 270 nm, or 281 nm, respectively. As shown in Figures 8A–D, LUT, SIT, MYA, and VAN all could be detected in YFBP. Also, HPLC analysis showed that the content of LUT, SIT, MYA, and VAN in YFBP was 0.08, 0.13, 0.09, and 0.05%, respectively.

### YFBP active ingredients inhibit proliferation, migration, and invasion of colorectal cancer cells and promote apoptosis

**FIGURE 9:**
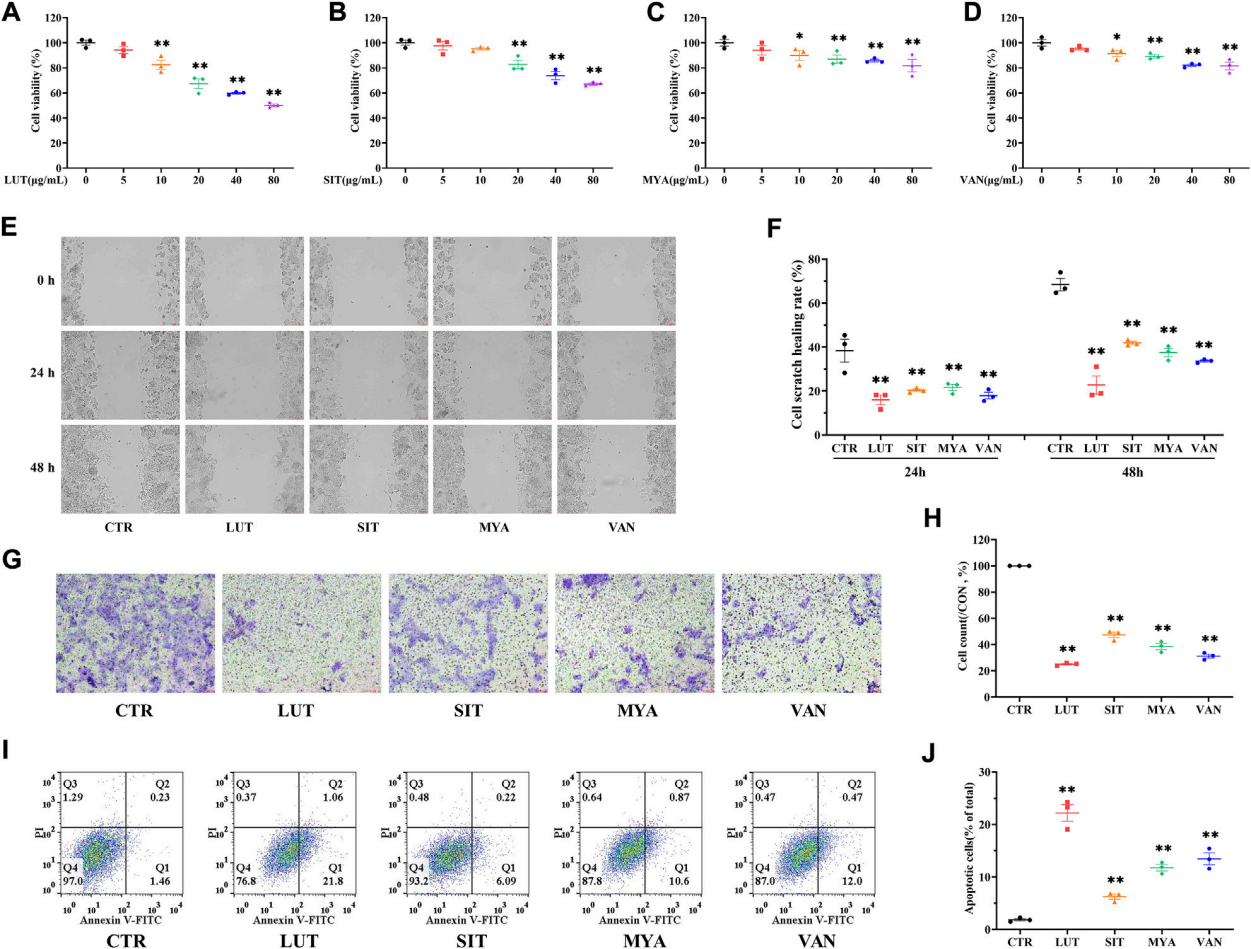
YFBP active ingredients inhibit proliferation, migration, and invasion of colorectal cancer cells and promote apoptosis. **(A–D)** Viability assay of LUT, SIT, MYA, or VAN. **(E ,F)** Cell scratch assay of LUT, SIT, MYA, or VAN. **(G ,H)** Cell invasion assay of LUT, SIT, MYA, or VAN. **(I,J)** Cell apoptosis assay of LUT, SIT, MYA, or VAN. Quantitative results are shown as the mean ± SEM from three independent experiments. ^*^ *p* < 0.05, ^**^ *p* < 0.01 vs. the CTR group.

The effect of LUT, SIT, MYA, or VAN (5–80 μg/ml) on HT-29 cell viability was evaluated using a CCK-8-based viability assay after incubating the cells with LUT, SIT, MYA, or VAN for 24 h. As shown in Figures 9A–D, LUT (10–80 μg/ml), SIT (20–80 μg/ml), MYA (10–80 μg/ml), and VAN (10–80 μg/ml) significantly suppressed the cell survival rate. Thus, 20 μg/ml of LUT, SIT, MYA, or VAN was used in the subsequent experiments. As shown in Figures 9E–H, cells treated with LUT, SIT, MYA, or VAN had a lower wound healing rate and level of invasiveness than control cells. As shown in Figures 9I,J, the proportion of apoptotic cells in the LUT, SIT, MYA, and VAN groups was significantly higher than that in the CTR group. The aforementioned studies show that LUT, SIT, MYA, and VAN may play an anti-tumor role in CRC treatment.

### YFBP active ingredients inhibit xenograft tumor growth

**FIGURE 10:**
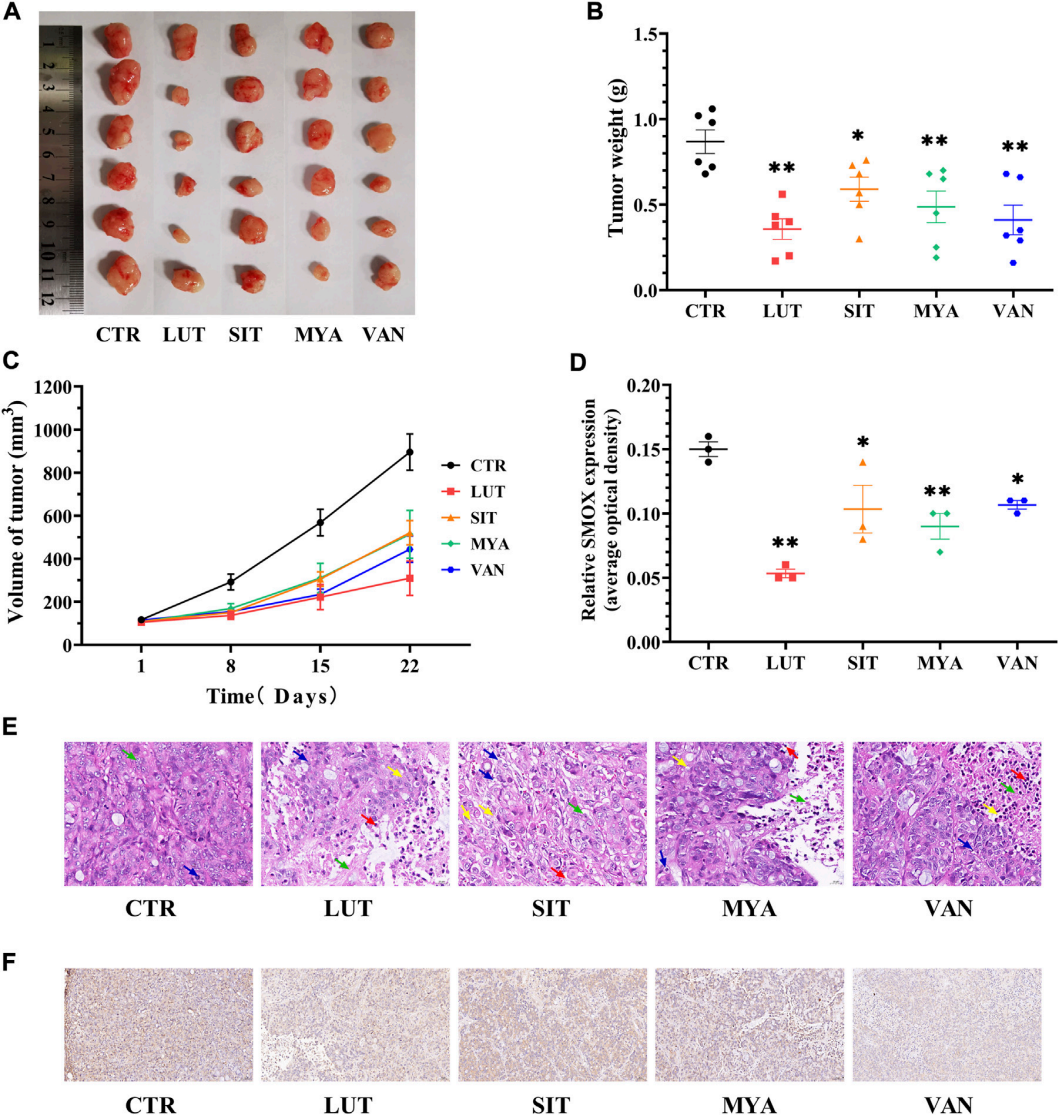
YFBP active ingredients inhibit *in vivo* HT-29 cell xenograft tumor growth. **(A)** Pictures of tumors taken after final treatment. **(B)** Tumor weight measured after final treatment. **(C)** Tumor size recorded during treatment. **(E)** HE staining of tumor. Scale bar = 20 μm. The green arrow indicates tumor cell necrosis, the blue arrow indicates nuclear fragmentation, the yellow arrow indicates cytoplasmic vacuolization, and the red arrow indicates neutrophil infiltration. **(D ,F)** Immunohistochemistry for SMOX. Scale bar = 50 μm. Quantitative results are shown as the mean ± SEM from three independent experiments. ^*^ *p* < 0.05, ^**^ *p* < 0.01 vs. the CTR group.

Tumors were isolated at the end of the mouse experiments, representative tumors were photographed, and tumor weights were quantitated. The tumor volume was calculated once a week until the end of the experiment (3 weeks). Tumor size (Figure 10A), weight (Figure 10B), and volume (Figure 10C) and histological aggressiveness were greatly reduced by treatment with LUT, SIT, MYA, or VAN. As shown in Figure 10E, in the CTR group, there were few necrotic areas in the tumor tissue. In the growth area, the tumor cells grew vigorously, the number of cells was large, the arrangement was dense, and the pathological mitotic figures were more common. Compared with the CTR group, the necrotic area in the tumor tissue of the LUT, SIT, MYA, or VAN group was significantly increased, the necrotic area was expanded, the arrangement of tumor cells in the growth area was loose, the number of cells was significantly reduced, the cytoplasm was vacuolated, and there were different degrees of inflammatory cell infiltration and fibrous tissue hyperplasia, suggesting that the growth of tumor cells in the drug group was significantly inhibited. As shown in Figures 10D,F, LUT, SIT, MYA, and VAN treatment suppressed SMOX gene expression. Collectively, these results further proved that the active ingredients of YFBP were effective against tumor tissues, as LUT, SIT, MYA, and VAN administration reduced the size, weight, and volume of tumors in the tumor formation assay in nude mice.

### YFBP active ingredients decreased SMOX production and downregulated SMOX expression

**FIGURE 11:**
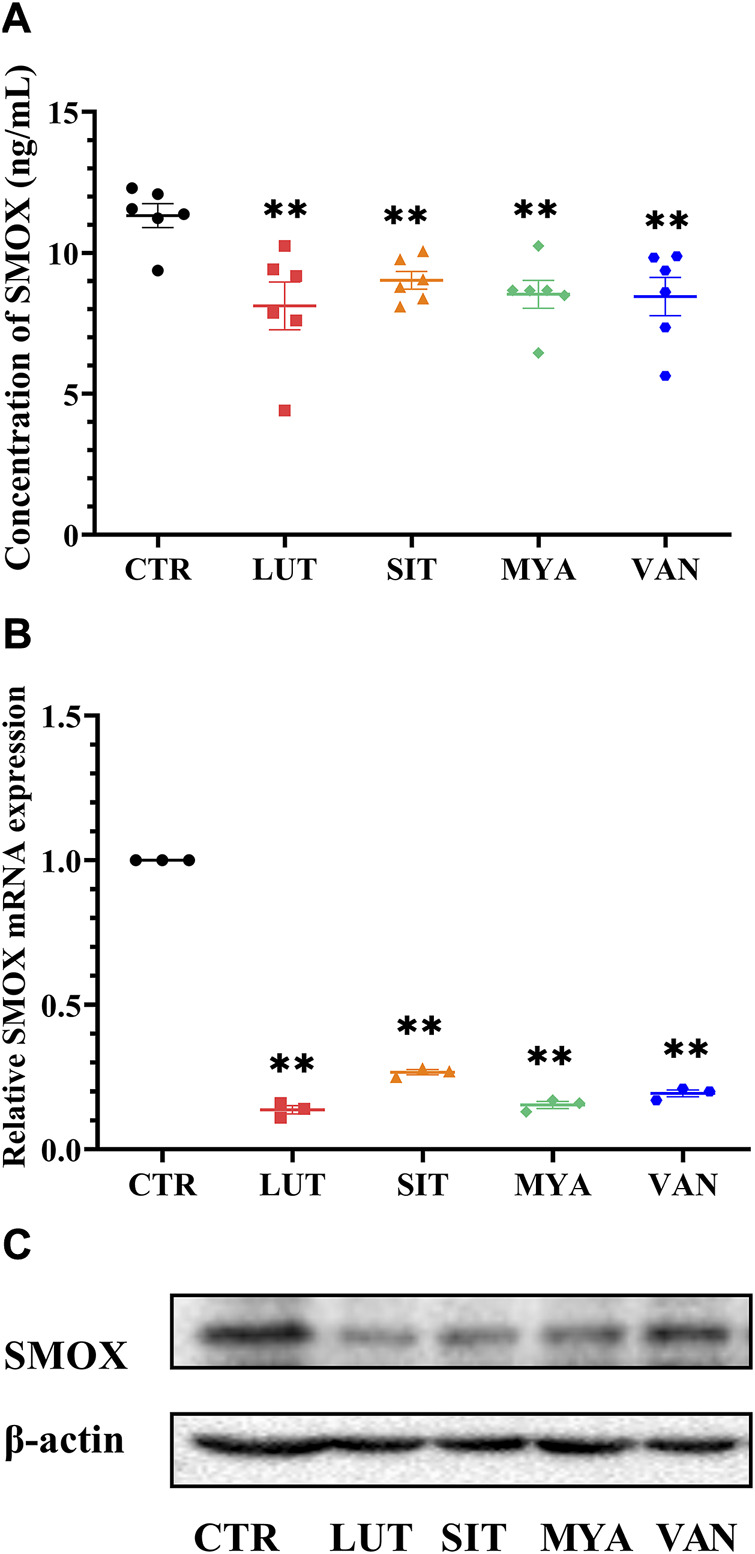
Effect of YFBP active ingredients on SMOX production and the expression of SMOX. **(A)** Level of SMOX was measured using ELISA kits. **(B)** mRNA expression of SMOX in the tumor tissue was measured by qRT-PCR. **(C)** Protein expression of SMOX in the tumor tissue was measured by Western blotting. Quantitative results are shown as the mean ± SEM from three independent experiments. ^**^ *p* < 0.01 vs. the CTR group.

The effect of the YFBP active ingredients on SMOX production was determined by ELISA. LUT, SIT, MYA, and VAN significantly suppressed SMOX secretion (Figure 11A). The expression of SMOX in the tumor tissue was assessed by quantitative real-time PCR at the same time points as the expression of SMOX protein by Western blotting. As shown in Figures 11B,C, LUT, SIT, MYA, and VAN suppressed SMOX gene expression, as well as SMOX protein expression.

### YFBP active ingredients decreased the expression of the inflammatory mediator

**FIGURE 12:**
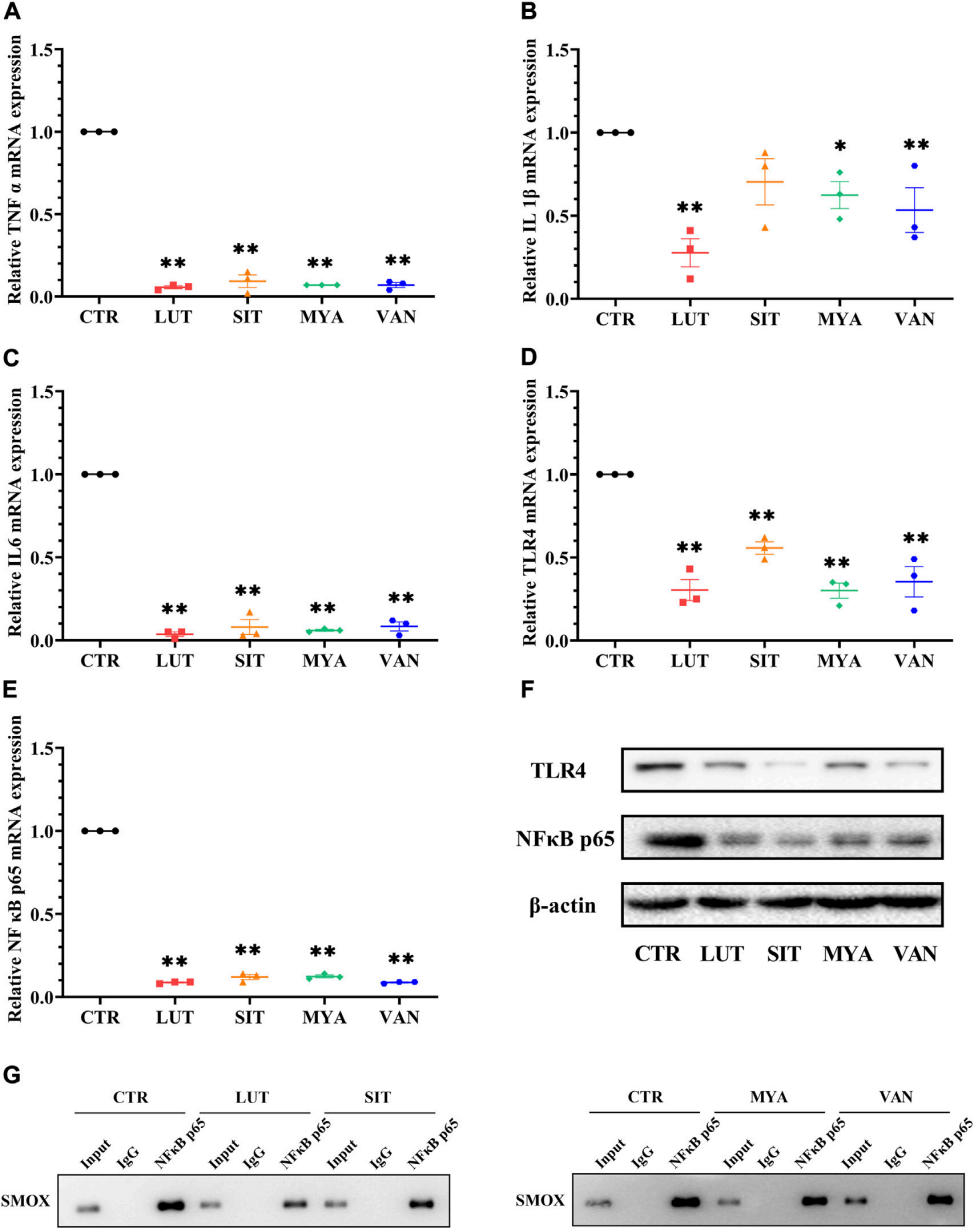
Effect of YFBP active ingredients on the expression of the inflammatory mediator and the regulation of inflammatory signaling. **(A–E)** mRNA expression of TNFα, IL-1β, IL-6 TLR4, and NF-κB p65 in the tumor tissue was measured by qRT-PCR. **(F)** Protein expression of TLR4 in the tumor tissue was measured by Western blotting. **(G)** Activation of transcription factor NF-κB p65 which binds to SMOX was measured by ChIP. Quantitative results are shown as the mean ± SEM from three independent experiments. ^*^ *p* < 0.05, ^**^ *p* < 0.01 vs. the CTR group.

To determine whether the anti-tumor effect of the active ingredients of YFBP is associated with the regulation of the expression of the inflammatory mediator, we assessed the gene expression of TNFα, IL-1β, and IL-6. As shown in Figures 12A–C, LUT, SIT, MYA, and VAN treatment suppressed the gene expression of TNFα, IL-1β, and IL-6. We next evaluated the gene expression of TLR4 and NF-κB p65. The results showed that LUT, SIT, MYA, and VAN treatment suppressed the gene expression of TLR4 and NF-κB p65 (Figures 12D,E).

### Effects of YFBP active ingredients on inflammatory signaling

Increasing evidence suggests that TLR4 and NF-κB play important roles in mediating inflammatory signaling pathways. To understand the mechanisms underlying the anti-inflammatory effects of the active ingredients of YFBP, the protein expression levels of TLR4 and NF-κB p65 were measured. TLR4 and NF-κB p65 proteins were significantly attenuated by pre-treatment with LUT, SIT, MYA, and VAN (Figure 12F). These results indicated that these active ingredients decreased the protein expression of TLR4 and NF-κB in the tumor tissue.

### ChIP assay on the activation of transcription factor NF-κB p65 which binds to SMOX

As shown in Figure 12G, Western blot analysis was performed to confirm the successful immune precipitation of the transcription factor NF-κB p65 which binds to SMOX in colorectal cancer cells. The results of ChIP show that the transcription factor NF-κB p65 may bind to SMOX in HT-29 colorectal cancer cells. Non-specific IgG was used as the negative control. The activation of transcription factor NF-κB p65 which binds to SMOX was significantly attenuated by pre-treatment with LUT, SIT, MYA, and VAN.

## Discussion

CRC is the third most common cancer worldwide, and inflammation is an important risk factor for its development and progression (Mantovani et al., 2008; Parang et al., 2017). Chronic inflammation can promote the development and progression of the disease and is considered a key predisposing factor for CRC in patients with inflammatory bowel disease (Tabung et al., 2018). Intestinal barrier injury may increase intestinal permeability to bacterial endotoxins, such as LPS, subsequently increasing mucosal inflammation and leading to systemic inflammation, thus increasing the risk of CRC (Natividad et al., 2018). Some studies have shown that the concentration of LPS is increased in tumor tissue and lymph node metastases in patients with CRC and that different regulatory factors of intestinal microbiota and their metabolites, including diet, antibiotics, probiotics, prebiotics, and fecal microbiota transplants, can prevent CRC occurrence and progression (Wong and Yu, 2019; Perillo et al., 2020). In recent years, major strides have been made in the diagnosis and treatment of CRC (Zhu et al., 2022). Despite these therapeutic achievements, many patients are still at risk for postoperative recurrence and metastasis; therefore, there is an urgent need for better exploration of the molecular biological characteristics and pathogenesis of CRC, instrumental for the development of novel preventive strategies. Aberrant overexpression of genes that determine the biological behavior of tumors is often associated with the development of malignancies and tumorigenesis, and the study of potential aberrantly expressed genes has significant implications in the diagnosis and treatment of CRC.

The composition of traditional Chinese medicine compounds is complex. It is not enough to study the extraction process only on a single component. At present, there are few studies on the extraction process and active ingredient analysis method of YFBP. The research indicated that Aconiti Lateralis Radix Praeparata has certain toxicity, and in clinical practice, Aconiti Lateralis Radix Praeparata was generally decocted first or for a long time and then mixed with Herba Patriniae for extraction (Zhou et al., 2016). Coicis Semen contains a large amount of starch, and its powder is easy to gelatinize when decocting. Coicis Semen powder may be directly used as medicine or wrapped with gauze and then decocted alone to avoid the influence on the extraction of Aconiti Lateralis Radix Praeparata and Herba Patriniae. In this study, 33 active components of YFBP were found to play an important role in CRC, affecting a variety of proteins and signaling pathways. The results show that the active components may have significant potential research value. Some of the 33 active components extracted from YFBP have been extensively studied previously and have been proven to have beneficial effects toward treating inflammation and cancer. Despite some being studied, the exact mechanism of the anti-tumor effect of LUT, SIT, MYA, or VAN is not clear. LUT, SIT, MYA, or VAN may have great potential research value (Selvendiran et al., 2006; Xu et al., 2016; Ramadoss and Sivalingam, 2020). In this study, luteolin, β-sitosterol, myristic acid, and vanillin were selected from the 33 active ingredients for further study experimentation and were found to have anti-tumor effects on CRC in a mouse model.

Network pharmacology provides a new method to understand the complex pharmacological mechanisms of traditional Chinese medicine (Hopkins, 2008). Owing to the multi-component and multi-target characterization of traditional Chinese medicine, it is difficult to comprehensively study. Network pharmacology can circumvent these complications, providing new strategies for drug development (Tang et al., 2020). In this study, the pharmacological mechanism of the action of YFBP against CRC was explored. As shown by the YFBP-CRC potential target gene network, many target genes appeared to be regulated by a variety of compounds in YFBP, including but not limited to AKT1, MAPK3, TLR4, HIF1A, and CASP3.

The results of bioinformatics analyses indicated that SMOX is highly expressed in CRC and is related to poor prognosis. SMOX is an enzyme containing flavin adenine dinucleotide that catalyzes the oxidative degradation of polyamine spermine to produce spermidine, hydrogen peroxide, and 3-aminopropionaldehyde. The use of polyamines and their metabolites as cancer biomarkers is not new, as this process is necessary for cell proliferation. Many cancers, especially oncogene-driven cancers, may be sensitive to interference from polyamine metabolism (Casero et al., 2018). The discovery that polyamines are directly involved in a variety of carcinogenic and cellular signaling pathways provides a new intervention point for cancer treatment (Casero et al., 2018). These findings suggest that spermine and its related products are involved in CRC pathogenesis. However, spermine metabolism in CRC has not been fully studied. Further study in this area would be important to further understand CRC pathogenesis. Some oncogenes are involved in polyamine metabolism in various cancers. SMOX plays a significant role in driving key oncogenic processes, and its expression is high in certain types of cancers (Goodwin et al., 2011). Since high levels of SMOX lead to abnormal activation of PA-driven oxidative stress and tumorigenesis, the development of PA analogs that can selectively inhibit SMOX would be desirable (Di Paolo et al., 2019). These inhibitors may be valuable probes for the study of carcinogenic pathways and may serve as chemo-preventive agents (Murray Stewart et al., 2018). The aim of this study was to investigate the effects of the active ingredients of YFBP on SMOX production and its regulatory mechanism in CRC. The results show that luteolin, β-sitosterol, myristic acid, and vanillin may exert anti-tumor effects by downregulating SMOX expression.

SMOX is a highly inducible enzyme in the polyamine catabolism cascade, and its expression is upregulated by inflammatory cytokines (including TNF-α and IL-6) (Cervelli et al., 2010). Moreover, the imbalance of SMOX changes intracellular polyamine levels and is related to the progression of various human diseases (Snezhkina et al., 2016; Wu et al., 2020). In the present study, luteolin, β-sitosterol, myristic acid, and vanillin were found to downregulate the expression of inflammatory cytokines such as TNF-α, IL-1β, and IL-6.

Cancer is a chronic disease. When infection and inflammation promote tumor development, signal transduction mechanisms that affect the malignant transformation or factors related to cancer monitoring are involved (Medzhitov, 2001; Akira et al., 2006). NF-κB is a key mediator of innate immunity and inflammation and has become an important endogenous tumor promoter (Karin, 2006). NF-κB is essential in tumors, potential tumors, and in inflammatory cells. It acts downstream of microbial or tissue damage perception through toll-like receptors and MyD88 signaling pathways, as well as inflammatory cytokine TNF-α- and IL-1β-mediated signaling pathways (Mantovani et al., 2008). In the present study, the active ingredients of YFBP downregulated the expression of TLR4 and NF-κB. Together with the previous results, this indicates that luteolin, β-sitosterol, myristic acid, and vanillin may downregulate SMOX expression through anti-inflammatory effects and regulate the TLR4/NF-κB signaling pathway.

Compatibility plays an important role in traditional Chinese medicine. The basic principle of practice is the compatibility of different herbal components with different syndromes. Nevertheless, the different efficacies and potential mechanisms of “prescription compatibility” remain unclear (Shao et al., 2019). To study traditional Chinese medicine through systems biology, starting from a holistic perspective and examining the details of these complex systems, the curative effect of traditional Chinese medicinal compound preparations can be evaluated (Qiu, 2007). As natural combination drugs, Chinese herbal medicines have great therapeutic potential for CRC as well as other diseases, but further research is needed to evaluate the anti-tumor effects of the combinational treatment of the active ingredients of YFBP in CRC.

## Conclusion

YFBP provides obvious advantages in CRC treatment. Network pharmacology was used to study the biological functions, target genes, and signaling pathways impacted by the YFBP active components during the treatment of CRC. Furthermore, the results of network pharmacological analysis were experimentally validated. The present study revealed that the active ingredients of YFBP, such as LUT, SIT, MYA, and VAN, might exert anti-tumor effects by downregulating SMOX expression through anti-inflammatory effects and the regulation of the TLR4/NF-κB signaling pathway. These results aid in further elucidating the molecular mechanism of YFBP anti-CRC activity and providing a theoretical foundation for further anti-tumor research.

## Data Availability

The raw data supporting the conclusion of this article will be made available by the authors, without undue reservation.

## Abbreviations

YFBP: Yiyi Fuzi Baijiang powder TCMSP: Traditional Chinese Medicine Systems Pharmacology TCMID: Traditional Chinese Medicine Integrated Database HIT: HitPredict Database STITCH: Stitch database PPI: Protein-to-protein interaction GO: Gene Ontology KEGG: Kyoto Encyclopedia of Genes and Genomes CRC: Colorectal cancer SSAT: Spermine/spermidine N1-acetyltransferase APAO: Polyamine oxidase SMOX: Spermine oxidase ROS: Reactive oxygen species DDR: DNA-damage response FBS: Fetal bovine serum TNF-α: Tumor necrosis factor-α IL-1β: Interleukin-1β IL-6: Interleukin-6 QED: Quantitative estimate of drug-likeness CTR: Control group LUT: Luteolin group SIT: β-Sitosterol group MYA: Myristic acid group VAN: Vanillin group.
