# Supplementary figures and images for: Identification of the effect and mechanism of Yiyi Fuzi Baijiang powder against colorectal cancer using network pharmacology and experimental validation
Source: Front Pharmacol. 2022 Oct 24;13:929836. doi: 10.3389/fphar.2022.929836 (PMC9637639; doi:10.3389/fphar.2022.929836)

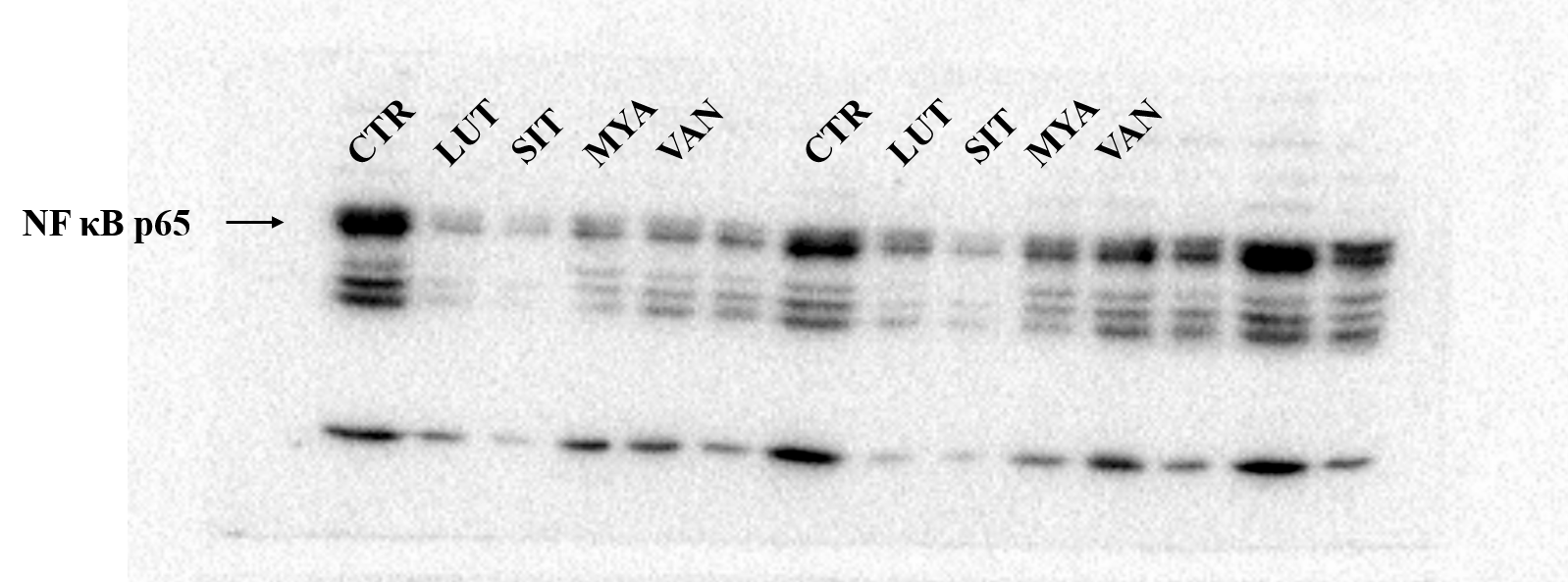

Supplement: Supplementary file 1 [file Image3.TIF]

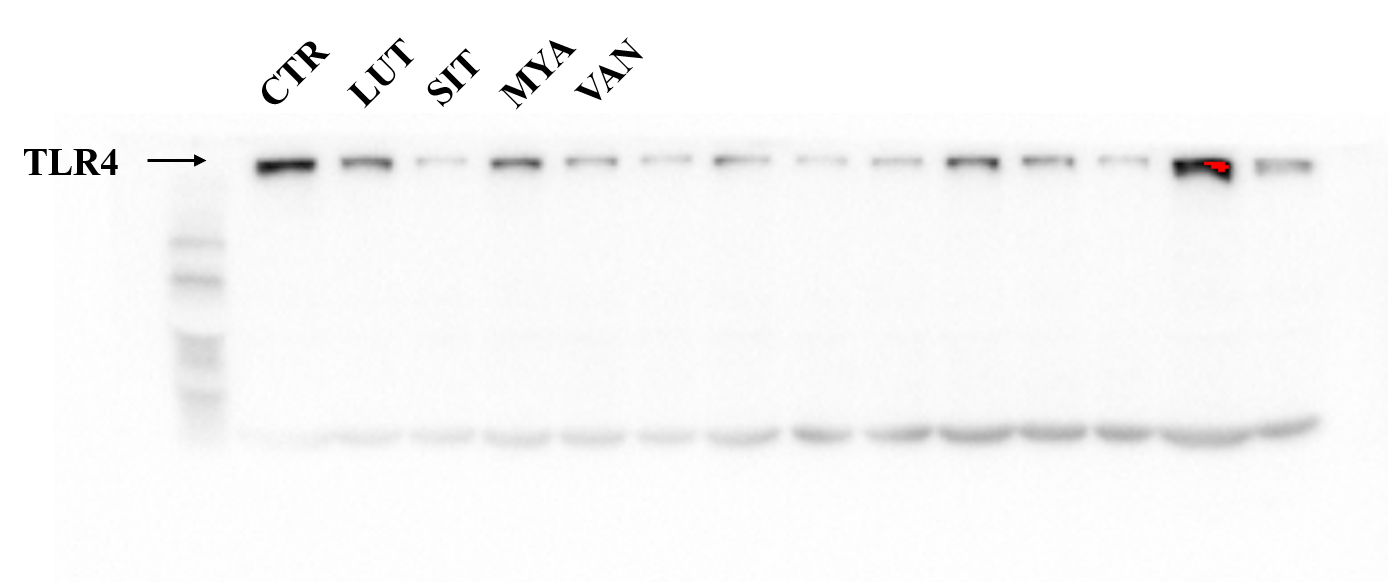

Supplement: Supplementary file 2 [file Image4.TIF]

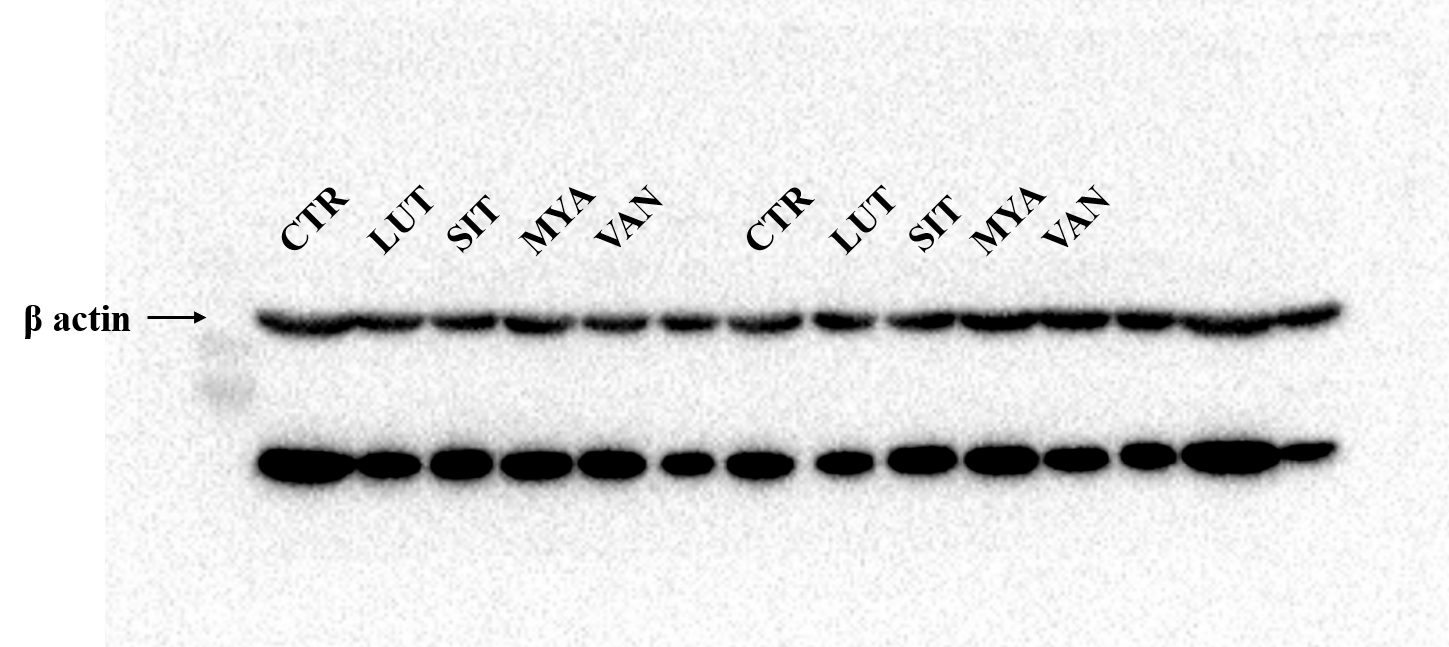

Supplement: Supplementary file 3 [file Image2.TIF]

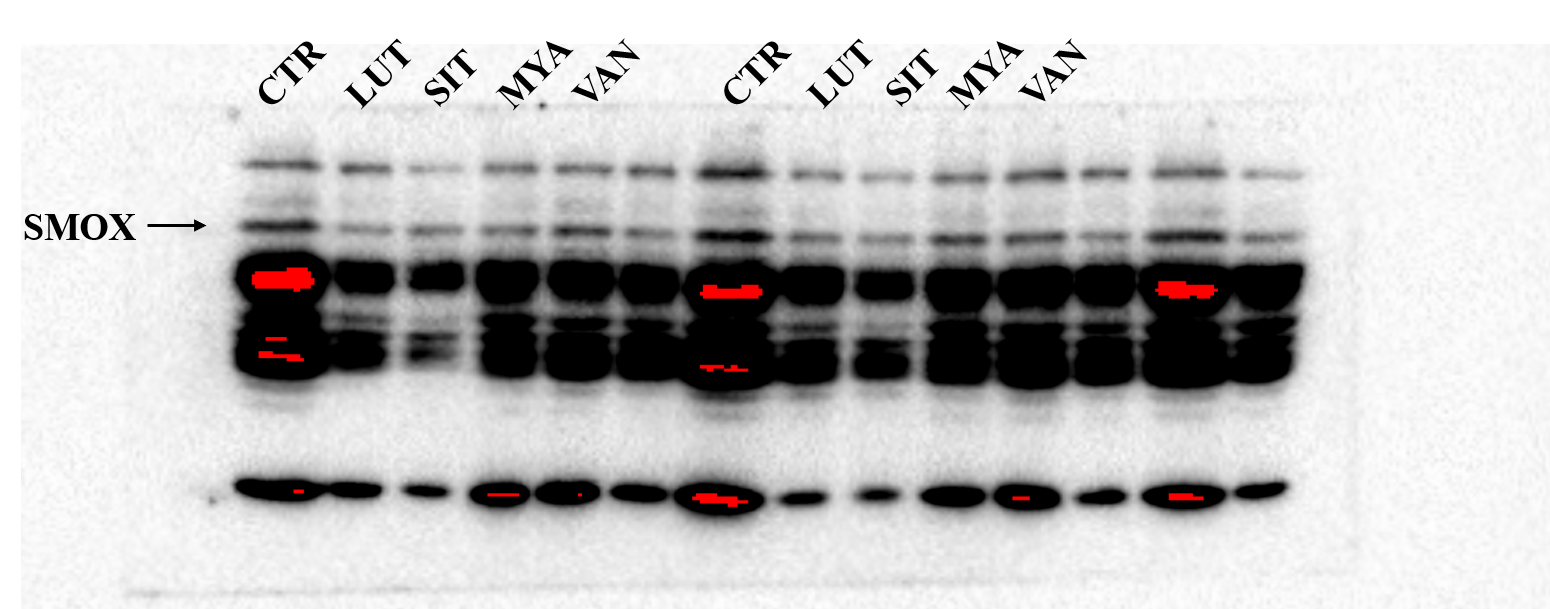

Supplement: Supplementary file 4 [file Image1.TIF]

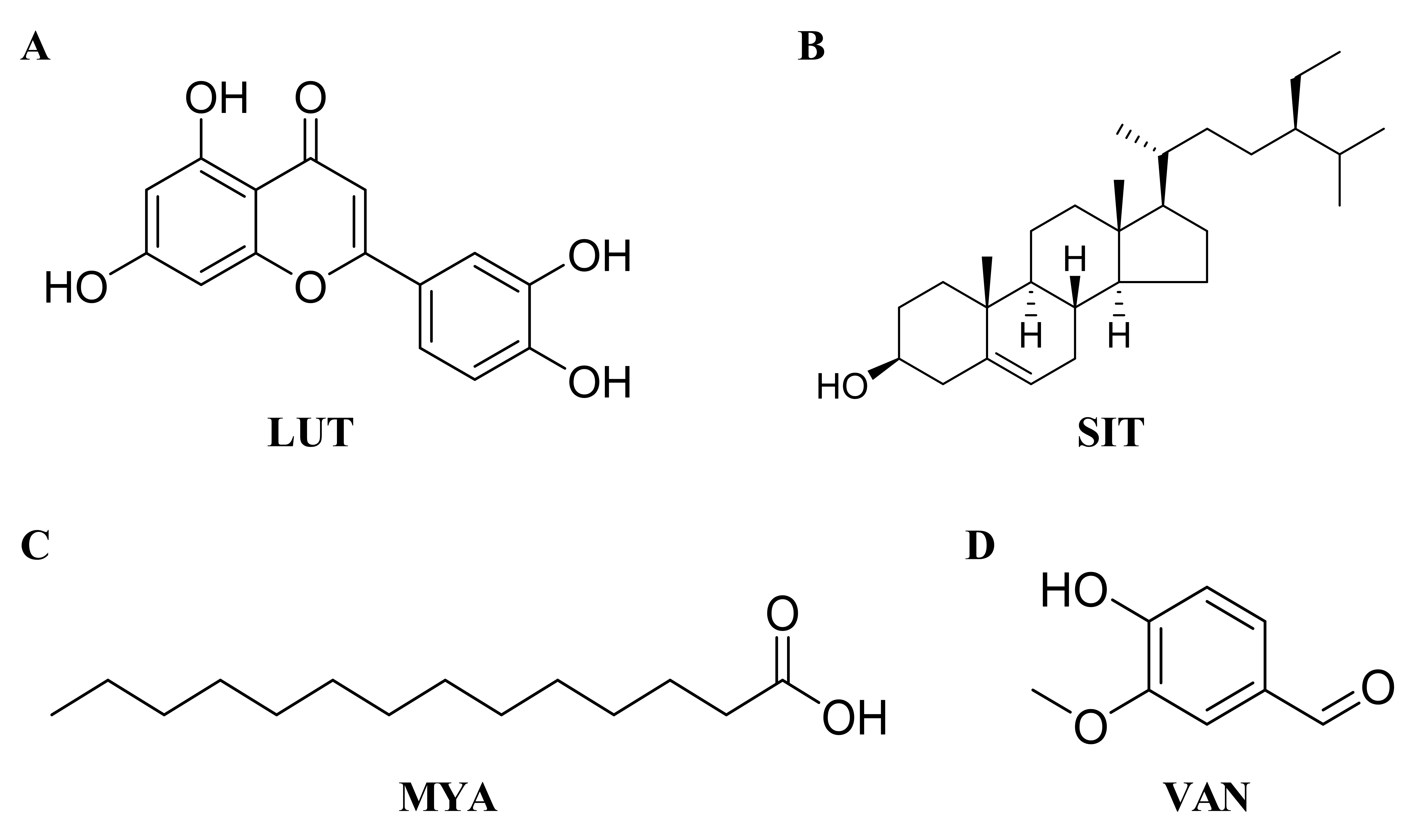

Supplement: Supplementary file 5 [file Image5.TIF]
